# Contribution of External and Internal Phosphorus Sources to Grain P Loading in Durum Wheat (*Triticum durum* L.) Grown Under Contrasting P Levels

**DOI:** 10.3389/fpls.2020.00870

**Published:** 2020-06-18

**Authors:** Mohamed El Mazlouzi, Christian Morel, Coralie Chesseron, Thierry Robert, Alain Mollier

**Affiliations:** ^1^ISPA, Bordeaux Sciences Agro, INRAE, F-33140, Villenave d’Ornon, France; ^2^Univ. Bordeaux, UMR 1391 ISPA, F-33000, Bordeaux, France

**Keywords:** durum wheat, phosphorus, ^32^P tracer, grain P concentrations, post-anthesis P uptake, P remobilization, hydroponic conditions

## Abstract

Phosphorus (P) in durum wheat grains after anthesis originates from either the external P source or the internal remobilization of P from different plant organs. The supply of P and its use by the plant are important factors that can affect the contribution of each source to grain P nutrition. Thus, this experiment aimed to quantify the origin of P in grains of durum wheat plants with different P nutritional status. Wheat plants were grown from juvenile stages to maturity in complete nutrient solutions with either high (0.125 mM) or low (0.025 mM) P concentrations in greenhouse conditions. Phosphorus in nutrient solutions was spiked by introducing ^32^P after anthesis to quantify the external P uptake and its partitioning within plant organs (spikelets, leaves, stems, roots, and post-anthesis tillers) and grains. Phosphorus use efficiency in durum wheat plants was also determined. The low and high P supply resulted in two highly different plant nutritional P status. Plants with low P status remobilized most of their stored P in all organs and allocated more than 72% of post-anthesis P uptake to grain P nutrition, whereas in the high P plants this was only 56%. Enhanced remobilization of P and the efficient allocation of newly acquired P to grains were crucial for durum wheat grain P nutrition grown under low P supply. The remobilization of P represented 81% of grain P in low P plants while it represented 65% for high P plants. Organs that contributed the most to P remobilization in low P plants were spikelets (43%) and leaves (35%). The post-anthesis tiller development was reduced in low P plants suggesting a preferential allocation of P to grains under this treatment. We concluded that P loading into grains in durum wheat is mainly derived from the remobilization of internal P sources stored before anthesis, even at high external P supply during grain filling.

## Introduction

Phosphorus (P) in soils is one of the most limiting factors for plant growth (Raghothama, 1999). In the conventional farming system, the application of P fertilizers is required to achieve optimal crop yield (Tilman et al., 2002). Phosphorus fertilizer management is mainly driven by the export of P in harvested products (Lott et al., 2000; Rose et al., 2013). Furthermore, P fertilizers are relatively inefficient, as only 20% of the applied P is taken by crops in the first year (Vance et al., 2003). This low efficiency is linked to a substantial negative impact associated with the transfer of P to the aquatic ecosystems (Brinch-Pedersen et al., 2002). In addition, unlike nitrogen, rock phosphate is a non-renewable resource that is subject to fluctuations in supply, which could be problematic to ensure future food security (Vance et al., 2003; Cordell and White, 2014). Given all these current challenges, improving P efficiency in cropping systems is of major interest. Achieving higher P efficiency is possible through a better understanding of the coordination of P uptake, transport, and remobilization in crops (Brinch-Pedersen et al., 2002; Rose et al., 2013; Wang et al., 2016).

Plants can adjust their metabolism in response to P availability (White and Hammond, 2008; Hawkesford et al., 2012). Although limited P supply might affect many physiological functions such as photosynthesis and energy transfer, it does not immediately lead to deficiency symptoms, except in cases of severe P limitation (Grant et al., 2001). This is because the internal remobilization of P from plant tissues could provide adequate amounts of P to ensure the growth of new sinks (Veneklaas et al., 2012). For cereals such as wheat, limiting P conditions reduce grain yield by limiting the number of productive tillers before anthesis (Horst et al., 1993). This strategy lowers the plant P requirement for biomass and grain production (Horst et al., 1993). Furthermore, low P requirement to produce biomass is associated with P utilization efficiency (PUE, defined here as biomass increase per unit of accumulated P) that was identified as an important target for crop improvement (Manske et al., 2002; Rose and Wissuwa, 2012). Crops with high PUE have typically lower tissue P concentrations and an optimized recycling and redistribution of P within the plant (White and Hammond, 2008; Veneklaas et al., 2012).

Phosphorus in grains is mainly stored in the form of phytate and play a crucial role in supplying P for seedlings before root development (Raboy, 2009; Nadeem et al., 2011; White and Veneklaas, 2012). However, the concentrations of P in grains are often over the need for germination and plant establishment (Rose et al., 2012; Pariasca-Tanaka et al., 2015; Wang et al., 2017). For example, Julia et al. (2018) reported that rice growth and development was unaffected by seed P concentration at seedling stage because root P uptake commences at the very earliest stages of plant development (two days after germination). Thus, lowering P concentration in grains would reduce the P export and thus contribute to the long-term sustainability of cropping systems (Wang et al., 2016; Julia et al., 2018). Prior to anthesis, wheat plants can accumulate large amounts of P in their organs (Grant et al., 2001; Veneklaas et al., 2012). After satisfying P demand created by different processes, the remaining P might be stored in vacuoles which could contain up to 90% of cellular P at adequate P supply (Raghothama, 1999). Its role is to maintain P homeostasis in the cytoplasm by adjusting the magnitude of P concentration fluctuations caused by different metabolic activities (Raghothama, 1999; Hawkesford et al., 2012). However, the growing grains become the main sink of P during the post-anthesis period. Plant organs could serve as a reservoir for P that can be remobilized to the developing grains (Dissanayaka et al., 2018; El Mazlouzi et al., 2020). Thus, the main sources of grain P are the internal P remobilized from different senescing plant organs and post-anthesis P uptake (Grant et al., 2001).

Wheat grain P is subject to genetic variability and depends on environmental conditions such as P and water availability (Batten, 1992; Manske et al., 2002). For instance several studies have shown that low P supply increases the proportion of remobilized P to grains (Batten, 1992; El Mazlouzi et al., 2020). Until now, the mechanisms of P uptake and transport during early plant growth are well characterized (Nadeem et al., 2011; Aziz et al., 2014). However, studies on the post-anthesis P fluxes in wheat are scarce. Although considerable progress has been made in understanding the processes controlling P fluxes from vegetative to reproductive organs, the mechanisms involved in P loading into grains are, however, not fully understood (Wang et al., 2016; Dissanayaka et al., 2018).

The objective of this study was therefore to quantify the contribution of external and internal P sources to the grain P nutrition in durum wheat plants with contrasted P supplies. Our working hypothesis was that plants grown with high P supply are expected to have a large proportion of P stored in different plant organs and would rely on these internal sources to supply the grain with P. In contrast, plants grown under low P supply are expected to maintain P uptake and optimize P remobilization to fulfil the grain P requirement. Therefore, plants of the latter group would depend on both external post-anthesis P uptake and a high P remobilization efficiency. The contribution of external P and internally remobilized P as sources for grain P nutrition can be quantified precisely using a P tracer. Thus, we performed a ^32^P-labelling experiment from anthesis to maturity to evaluate the contribution of both sources to grain P nutrition in durum wheat.

## Materials and Methods

### Experimental Set-Up, Plant Growth, and P Treatments

**Figure 1:**
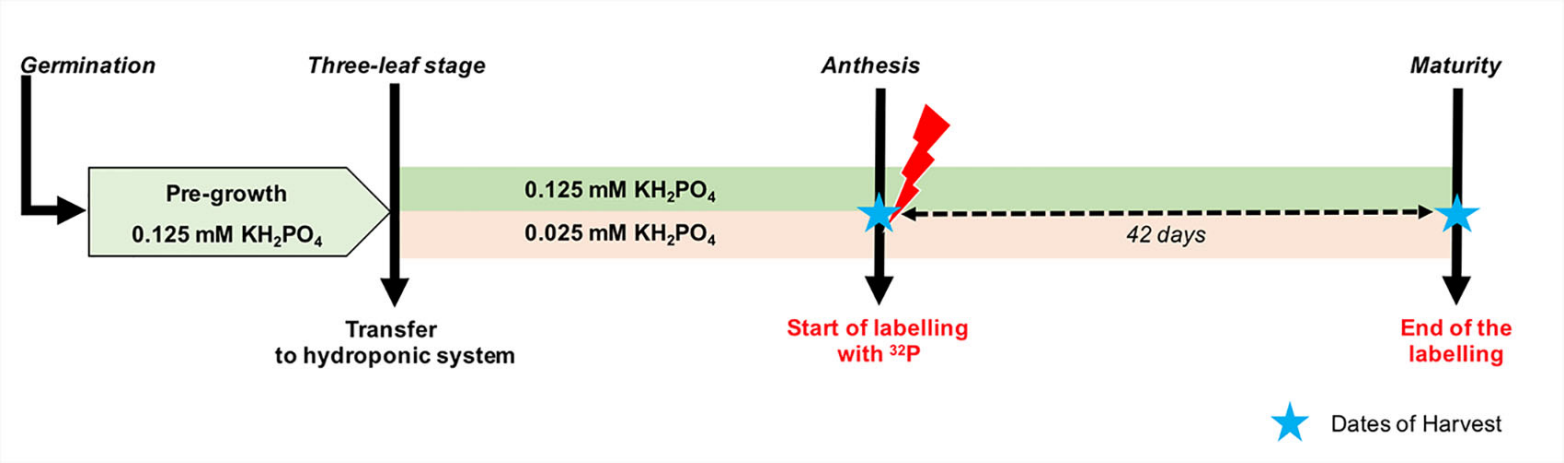
Schematic overview of the experimental design used in the experiment. After third leaf emergence, durum wheat plants were grown in a nutrient solution with two levels of P supply. Immediately after anthesis, three plants of each treatment were labeled with ^32^P until maturity. The dashed arrow indicates the labelling period and stars indicate the dates of harvests. Growth conditions (temperature and relative humidity) are presented in **Supplementary Figure 1**.

Hydroponic culture conditions were similar to those previously described by El Mazlouzi et al. (2020). Briefly, seeds of *Triticum durum* cv. *Sculptur*, a spring durum wheat cultivar widely cultivated in France, were disinfected in 6% (v/v) sodium hypochlorite (H_2_O_2_) solution and then germinated on moist paper and kept in darkness for 3 days at 25°C. After germination, seedling were transferred to 50-ml Falcon^®^ tubes filled with a modified Hoagland nutrient solution. Subsequently, 15-day-old plants of a similar size were selected and transplanted to black plastic pots (1 plant per pot) containing 5.5 L of modified Hoagland nutrient solution. The nutrient solution had the following initial composition: 0.625 mM KNO_3_, 0.85 mM KCl, 1.25 mM Ca(NO_3_)_2_, 0.5 mM MgSO_4_, 46.25 *μ*M H_3_BO_3_, 1 *μ*M MnCl_2_, 10 *μ*M ZnSO_4_, 2 *μ*M CuSO_4_, 0.03 *μ*M (NH_4_)_6_Mo_7_O_24_, 100 *μ*M NaFe EDTA, 25 *μ*M HEDTA, 2 mM MES buffer, and 50 mg L^−1^ SiO_2_. P was supplied in nutrient solution from juvenile stages (three-leaf stage) to maturity as KH_2_PO_4_ at concentrations of 0.025 or 0.125 mM, designated hereafter in text as low and high P supply, respectively. These two levels of P supply were set to induce a differential plant P nutritional status (**Figure 1**). KCl was added to the low P supply to ensure the same potassium concentration for both treatments. The initial pH was adjusted to 6.0 ± 0.5 using solid KOH. The nutrient solution was mixed by air-bubbling and refreshed continuously by an overflow-type system supplying 6% of fresh nutrient solution per day. After tiller emergence and until anthesis (2 weeks), the renewal rate was increased up to 28% of nutrient solution per day for both low and high P supply.

Since high rates of pre-anthesis tillers are common in hydroponic culture, only the first four tillers were allowed to grow. This number of productive tillers is comparable to that found in the field for durum wheat, which ranges from two to four tillers in the absence of any limiting factors (Elhani et al., 2007). Tillers developed after anthesis were allowed to grow during the labelling period (see next section). The pots were arranged in a randomized block design with three replicates of each treatment.

Plants were grown in a greenhouse under a day/night cycle of 16/8 h. The average air temperature and relative humidity were 17°C and 64%, respectively. Natural light was supplemented with LED lamps (model LED, Ledlyt, France) providing an average of photosynthetic photon flux density of 450 *μ*mol photons m^−2^ s^−1^. Air temperature and relative humidity during the growing period are presented in the **Supplementary Figure 1**.

Three replicates of each P treatment with synchronized plant development were harvested at anthesis to determine their P status. Three other replicates were selected for the ^32^P-labelling experiment and harvested at maturity.

### ^32^P-Labelling of Phosphate Ions in Nutrient Solutions

The labelling experiment was carried out in a greenhouse dedicated to handle radioisotopes. The environmental conditions were similar to those described above. To prepare the labeled nutrient solution on the day of anthesis, 24 L of the nutrient solution with the same composition was prepared for each treatment as mentioned earlier. Then, the low and high P nutrient solutions were spiked with about 8 and 4 MBq of H_3_^32^PO_4_ with specific radioactivity of 325 TBq/mmol (NEX054010MC, Perkin Elmer). Three replicates of one wheat plant were transferred to pots containing 5 L of each labeled nutrient solution. Before changing the nutrient solution, the root systems of the wheat plants were carefully washed three times. Thereafter, because of experimental constraints and to limit exposure to radiation, nutrient solutions were not refreshed continuously but resupplied twice a week with the same labeled nutrient solution until harvest. The nutrient solution was aerated continuously. Before each resupply, the ^32^P-labeled nutrient solution was stirred thoroughly using a small water pump. The labelling period lasted for 42 days (**Figure 1**).

### Plant Sampling and Measurements

To ensure plant sampling homogeneity, heads that reached anthesis on the same day were tagged. At anthesis and 42 days after anthesis, plants (three replicates) were harvested and divided into the following organs: roots, stems, lower leaves, flag leaves, spikelets (including rachis), and grains. The tillers developed after anthesis were pooled. Roots were washed with deionized water three times to remove the adhered nutrient solution. Plant organs were then oven-dried at 60°C for 72 h and their dry weights were determined. The fresh weight of grain at maturity were determined to calculate the grain moisture content (GMC). In addition to grain yield, grain number per plant and thousand-grain weight were also determined.

### P Concentration in Plant Organs

The P concentration (mg g^−1^) in roots, stems, lower leaves, flag leaves, spikelets, grains, and pooled tillers was determined using the same procedures as described in El Mazlouzi et al. (2020). Briefly, sub-samples of plant material were ashed at 550°C for 5 h. The ashes were dissolved in 2.5-ml nitric acid and placed on a hotplate to evaporate. After cooling, the mineralized samples were filtered and diluted to 50 ml in ultra-pure water. Phosphorus concentration (mg g^−1^) was then determined using the malachite green colorimetric method (Van Veldhoven and Mannaerts, 1987). The P amount in plant organs was calculated by multiplying their respective P concentrations by dry weight. The P amount in the whole plant (mg P plant^−1^) was calculated as the sum of P amount measured in each organs.

### Determination of ^32^P Activity and Parameters of Biomass and P Partitioning

A sub-sample of 1 ml of each mineralized and filtered samples and 3 ml of scintillation cocktail for aqueous solution (Insta-gel Plus Packard, Perkin-Elmer, Waltham, Massachusetts, USA) were placed in a glass scintillation vial (Simport™ Scientific S207). Thereafter, the ^32^P-activity in filtered samples were measured using a liquid scintillation counter (TriCarb 2100, Packard). All ^32^P countings were corrected for the radioactivity decay.

External P uptake from the nutrient solution was quantified by applying isotopic dilution principles and assuming that no ^32^P/^31^P-fractionation occurred during external P uptake by roots and P transport within the plant. Considering that R/P*_ns_* is the specific activity measured in the nutrient solution, and r is ^32^P-activity measured in the mineralized samples, then the following calculations can be used to calculate the amount of P in each organ that derives from the external nutrient solution (P*_ext_*, mg P plant^−1^) (Morel and Fardeau, 1990):

$$\frac{R}{P_{ns}}=\frac{r}{P_{ext}}=>P_{ext}=\frac{r}{\left(\frac{R}{P_{ns}}\right)}$$

where R and P*_ns_* are the amount of ^32^P and ^31^P measured in the nutrient solution, respectively. Thus, post-anthesis P uptake is the sum of external P measured in all plant organs.

The total amount of remobilized P was calculated by subtracting the amount of P originating from post-anthesis P uptake in net sink organs (grains and post-anthesis tillers) from the whole plant P amount. However, the amount of remobilized P from each plant organ that acted as a source of P (spikelets, leaves, stems, and roots) was calculated as the difference in P amount between anthesis and maturity. All these fluxes were expressed as the percentage of P at the whole plant level. The efficiency of P remobilization (%) was calculated as (total P remobilization)/(total P anthesis)×100. The efficiency of P utilization (%) was calculated as the ratio of grain yield to the amount of P in the whole plant.

The shoot:root ratio was calculated as the ratio of total biomass in the aboveground parts and root biomass. Harvest index (HI, %) and P harvest index (PHI,%) were calculated as grain dry biomass or amount of P in grain as a percentage of the aboveground biomass or aboveground P, respectively. Although the post-anthesis tillers have produced spikes, these did not contain viable grains as they did not reach maturity and are therefore not included in the harvest index calculations.

### Statistical Analysis

Statistical analyses were undertaken using R statistical software version 3.4.4 (R core team, 2018). Data were expressed as mean ± standard error (SE) of three replicates. Significant differences between P treatment and harvest stages (anthesis vs. maturity) were determined separately using Student’s t test (*P* < 0.05).

## Results

### Plant Growth and Grain Characteristics

Durum wheat (cv. *Sculptur*) plant biomass increased from anthesis to maturity in both P treatments. Nevertheless, plants grown under low P supply produced fewer post-anthesis tillers than those grown at high P supply (*P* < 0.05), which led to significantly lower total biomass in these plants at maturity (**Table 1**). The biomass of spikelets, stems, and roots also increased while the biomass of leaves remained relatively constant throughout the post-anthesis period. At maturity, tiller biomass represented 28% of the total biomass at high P supply while it was only 4% at low P supply.

**Table 1:** Biomass (g), P concentrations (mg g^−1^), and P amount (mg) at anthesis and maturity of different durum wheat organs grown under high P (0.125 mM) and low P (0.025 mM) supply.

Stage	P Supply	Grain	Spikelets	Flag leaves	Lower leaves	Stems	Roots	Post-anthesis tillers	Whole plant
**Biomass (g)**									
Anthesis	High P	–	1.6 ± 0.1	0.88 ± 0.08	1.2 ± 0.07	1.8 ± 0.1	2.4 ± 0.3	–	7.9 ± 0.7
	Low P	–	1.6 ± 0.02	0.97 ± 0.02	1.3 ± 0.04	1.8 ± 0.06	2.1 ± 0.1	–	7.8 ± 0.2
Maturity	High P	11.3 ± 0.3	2.8 ± 0.2	0.99 ± 0.02	1.1 ± 0.06	3.5 ± 0.1	4.0 ± 0.1	9.5 ± 0.5	33.2 ± 1.1
	Low P	9.7 ± 0.5	3.1 ± 0.2	0.95 ± 0.02	1.1 ± 0.07	3.6 ± 0.1	3.3 ± 0.1*	0.9 ± 0.1***	22.7 ± 0.9**
**P concentration (mg g^−1^)**									
Anthesis	High P	–	5.4 ± 0.1	5.8 ± 0.3	6.4 ± 0.3	6.3 ± 0.5	4.1 ± 0.4	–	–
	Low P	–	3.6 ± 0.3*	2.7 ± 0.1**	1.7 ± 0.1**	1.3 ± 0.2**	1.5 ± 0.4*	–	–
Maturity	High P	3.8 ± 0.1	1.6 ± 0.3	2.1 ± 0.2	2.6 ± 0.2	1.2 ± 0.3	1.3 ± 0.2	1.4 ± 0.1	–
	Low P	1.8 ± 0.1***	0.3 ± 0.1*	0.4 ± 0.1*	0.3 ± 0.1**	0.2 ± 0.3	0.6 ± 0.1*	0.6 ± 0.1	–
**P amount (mg)**									
Anthesis	High P	–	8.9 ± 0.9	5.2 ± 0.7	7.4 ± 0.7	11.4 ± 1.5	9.8 ± 1.6	–	42.7 ± 5.4
	Low P	–	5.9 ± 0.4	2.6 ± 0.2	2.1 ± 0.1***	2.2 ± 0.3***	3.0 ± 0.1*	–	15.8 ± 0.9*
Maturity	High P	42.9 ± 0.7	4.7 ± 1	2.1 ± 0.2	2.9 ± 0.1	4.2 ± 0.9	5.1 ± 0.7	13.6 ± 1	75.5 ± 2.6
	Low P	17.3 ± 0.9**	0.9 ± 0.2**	0.4 ± 0.04*	0.4 ± 0.1**	0.7 ± 0.04*	2 ± 0.5	0.5 ± 0.1***	22.2 ± 0.9***

Values are means ± standard error of three replicates. For each stage, means followed by asterisk indicate significant difference between P treatment (*P < 0.05; **P < 0.01; ***P < 0.001).

**Figure 2:**
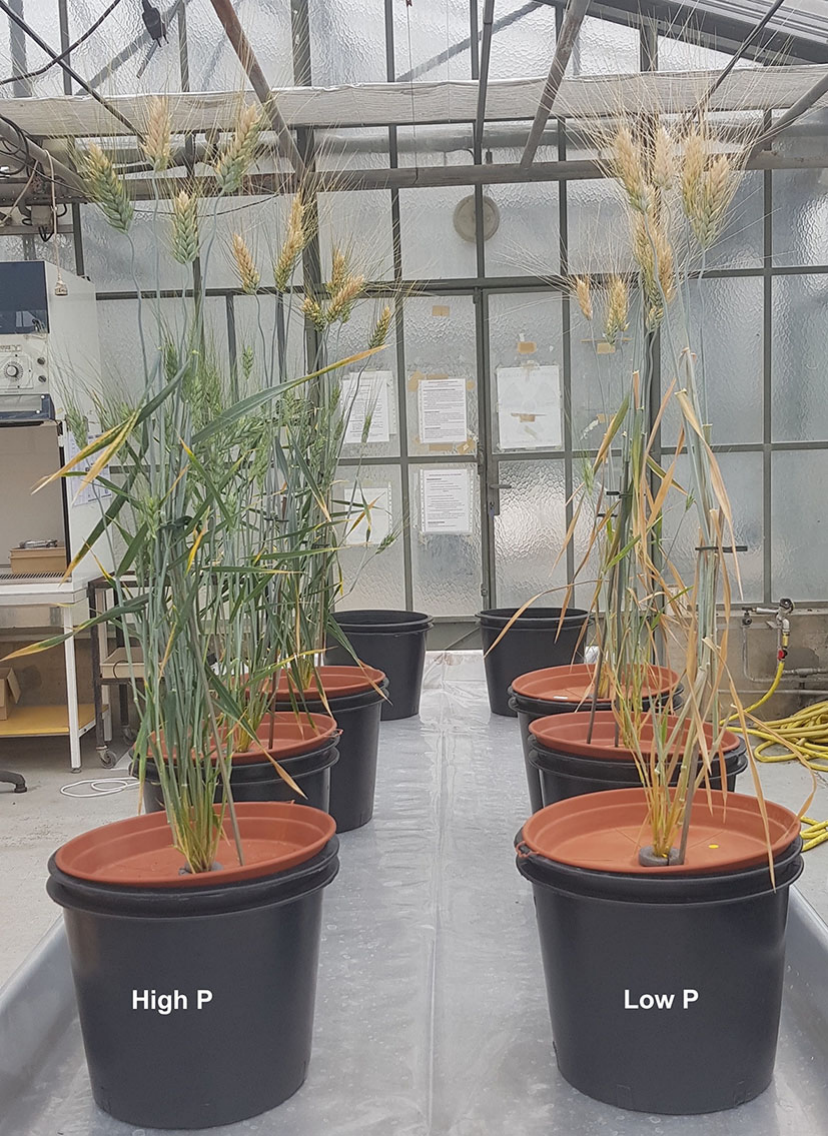
The phenotype of durum wheat plants (cv. *Sculptur)* at maturity. Plants were grown in nutrient solution with a high or low P supply from third-leaf stage to maturity.

Contrary to expectation, there was no significant effect of the low P supply on grain yield and grain number, but thousand grain weight significantly decreased (*P* < 0.05). The high variability in grain number obtained by each plant under low P supply might explain this result (**Table 2**). Low P plants invested more biomass into roots which resulted in lower shoot to root ratio in comparison to high P plants (5.9 ± 0.2 and 7.4 ± 0.1, respectively). The harvest index of low P plants were higher than those of high P plants. Accelerated leaf yellowing was observed at maturity for the plants grown under low P supply in comparison to high P supply plants (**Figure 2**). Consistent with the accelerated senescence, GMC was slightly lower in low P plants (High P: 40%, Low P: 36%). This means that durum wheat grains were harvested after physiological maturity stage.

**Table 2:** Grain number, thousand grain weight (TGW), grain moisture content (GMC), shoot:root-ratio, harvest index (HI), P harvest index (PHI), efficiency of remobilization, and efficiency of P utilization in high P and low P durum wheat plants.

	High P plants	Low P plants
Grain number (plant^−1^)	245 ± 4	237 ± 11
TGW (g)	46.1 ± 1.03	40.9 ± 0.4*
GMC (%)	40 ± 2	36 ± 2
Shoot:root ratio	7.4 ± 0.1	5.9 ± 0.2**
HI (%)	39 ± 1	50 ± 1**
PHI (%)	62 ± 3	86 ± 1**
Efficiency of P remobilization (%)	73.3 ± 2.8	82.1 ± 2.3
Efficiency of P utilization (%)	15 ± 0.9	43.7 ± 0.6**

Values are means ± standard error of three replicates. Significant difference between high P and low P plants are indicated by asterisks (*P < 0.05; **P < 0.01).

### P Concentrations and Partitioning Among Plant Organs

At both anthesis and maturity, P concentration in all durum wheat organs were significantly lower in low P plants compared with the high P plants (*P* < 0.05) (**Table 1**). From anthesis to maturity and regardless of P supply, P concentration decreased in all plant organs. At anthesis, the highest P concentration in plant organs was observed in leaves (6.4 ± 0.3 mg g^−1^) for high P supply plants, whereas it was observed in spikelets for low P supply plants (3.6 ± 0.3 mg g^−1^). The average P concentration in the grains was two-fold higher under high P supply than under low P supply (High P: 3.8 ± 0.1 mg g^−1^, Low P: 1.8 ± 0.1 mg g^−1^) (**Table 1**). The extent of change observed in P concentrations between anthesis and maturity in low P plants was different from that observed for high P plants. This extent was more pronounced for low P plants reaching concentration as low as 0.2 ± 0.3 mg g^−1^ in stems, for example, suggesting an enhanced P remobilization in those plants.

As expected, the high P plants accumulated a significantly greater amount of P than the low P plants (*P* < 0.05). Whole plant P amount increased from 42.7 ± 5.4 mg to 75.5 ± 2.6 mg and from 15.8 ± 0.9 to 22.2 ± 0.9 in high P and low P plants between anthesis and maturity, respectively (**Table 1**). The amount of P in grain and tiller increased during grain filling in high P plants while the grain was the only sink of P in low P plants. The amount of P in roots, stems, leaves and spikelets decreased in both P treatment during grain filling indicating net P remobilization from these tissues. However, P remobilization in low P plants was more pronounced than in high P plants leading to P amount as low as 0.4 mg P in flag leaves, for example.

At maturity, P partitioning between different plant organs was influenced by P supply. Under high P supply, grain P represented 57% of total P at whole plant levels while post-anthesis tillers represented 17%. In contrast, grain P represented 78% of total P under low P supply indicating a high rate of P allocation to grains. The P harvest index calculated as the ratio of P in grains to the aboveground P was significantly higher in low P plants in comparison to high P plants (**Table 2**).

Efficiency of P utilization (defined as the ratio of grain yield to whole plant P amount) was significantly lower in high P plants (15 ± 0.9%) compared to low P plants (43.7 ± 0.6%). The efficiency of P remobilization was slightly higher in low P plants but was not significantly different between high and low P supply.

### Post-Anthesis P Uptake and Remobilization of P From Organs

**Figure 3:**
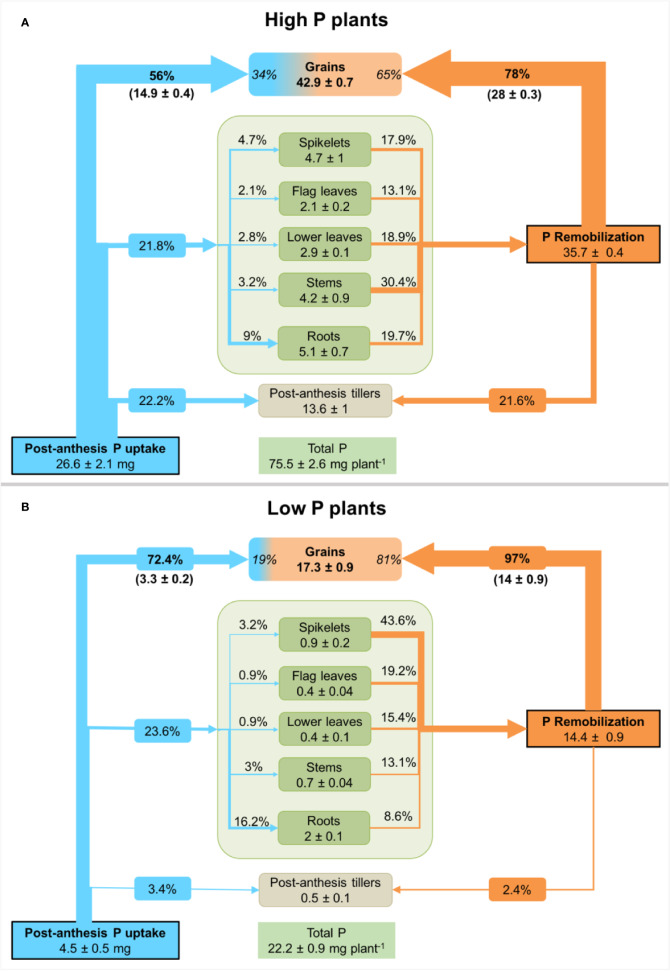
Net P fluxes in durum wheat plants grown under high P **(A)** or low P supply **(B)**. P fluxes are presented as a percentage of post-anthesis P uptake (blue arrows, determined from ^32^P-labelling) and remobilized P (orange arrows, calculated from the difference between P amount at anthesis and maturity) in different durum wheat plants organs. Values inside plant organs represent P amount (mg P) at maturity. Percentage values inside the grain compartment represent the contribution of post-anthesis P uptake and P remobilization to grain P. Values are means ± standard error of three replicates.

The calculations undertaken using ^32^P as a tracer showed that durum wheat plants had absorbed about 4.5 ± 0.5 mg P and 26.6 ± 2.1 mg P for low and high P supply from anthesis to maturity, respectively (**Figure 3**). This is equivalent to, respectively, 20% and 35% of total P uptake. In high P plants, 56% is recovered in grain and 22% in tillers. The remaining external P is allocated to roots, spikelets and leaves. In contrast, grains in low P plants recovered 72% of external P and the remainder is mainly distributed to roots (16%) and other organs, suggesting a tight regulation of P transport and translocation to grains.

All plant organs present at anthesis acted as a source of P as shown by the decline in their P amount at maturity. The total net P remobilized from these organs was 14.4 ± 0.9 mg P and 35.7 ± 0.4 mg P for low and high P plants, respectively. Stems, leaves and roots contributed to more than 82% of remobilized P in high P plants (**Figure 3A**). Net remobilization of P was observed in all plant organs. In low P plants, spikelets and leaves contributed to more than 78% of remobilized P (**Figure 3B**). The enhanced P remobilization contributed to fulfilling grain P requirement in both P treatments. In low P plants, P remobilization from different plant organs represented 81% of grain P while its represented 65% for high P plants.

## Discussion

Phosphorus loading into grains results from post-anthesis P uptake and the remobilization of internal P sources. Both sources contribute to the whole plant P use efficiency. Thus, understanding the relationship between these two components is valuable to engineer crops with improved P nutrition strategies. Tracer methods offer some advantages compared to the budget method that has been widely used to study nutrient fluxes at the whole plant level (Masclaux-Daubresse et al., 2008). They provide a better estimation of external P uptake and reduce uncertainties associated with inter-plant variability (Masclaux-Daubresse et al., 2008). In the present study, the use of a ^32^P-tracer enabled us to determine the partitioning of the newly acquired P during the post-anthesis period. It also allowed us to quantify the net contribution of internal P sources to the P allocated to grains at maturity in durum wheat (*Triticum durum* L.).

### Effects of P Supply on Plant Growth and P Accumulation

The lack of response to P supply in term of biomass production at the anthesis or in grain yield at maturity indicates that the levels of P supply to plants were sufficient for durum wheat growth under our experimental conditions. However, high P plants had accumulated large amounts of P in comparison to low P plants, which resulted in plants with highly different P nutritional status. Phosphorus concentrations were significantly higher in the plants grown at high P supply than at low P supply (**Table 1**). The leaf P concentration in low P plants at anthesis was slightly below the critical reported value for maximum yield of 3 mg P g^−1^ for wheat (Veneklaas et al., 2012). Nevertheless, this critical value could vary depending on species, genotype, and the supply of other nutrients (White and Hammond, 2008). It also appeared that high P plants accumulated much more P than what they require to produce optimum yield. Concentration as high as 6.4 mg P g^−1^ in lower leaves suggests the occurrence of P luxury consumption in high P plants. In addition, the production of high post-anthesis tiller number, which is uncommon under field conditions, could potentially have influenced the amount of carbon and P allocated to grains in high P plants as they represented 28% of total biomass at maturity. Our results show that this additional sink competed with grains for P and potentially for other nutrients. Bollons and Barraclough (1997) found that the main effect of low P supply on wheat growth was the decrease in the number and weight of tillers. In our study, since tillers were removed before anthesis, the total biomass was not different between treatment at this stage. However, after anthesis where tillers were allowed to grow, differences in biomass accumulation were related to the capacity of the plants to produce post-anthesis tillers. The P concentration in the grain of durum wheat plants (from 1.8 to 3.8 mg P g^−1^ for low and high P plants, respectively) in our experiment covers the range of variation of P concentration found in field studies for wheat (Batten, 1992; Manske et al., 2002). For example, grain P concentrations below 2 mg P g^−1^ are frequently associated with wheat plants that have been grown under limited P conditions (Batten, 1986). It is also noteworthy that yield parameters such as thousand grain weight and grain number per head are similar to those found under field condition for wheat (Elhani et al., 2007; Su et al., 2009).

### Contribution of External and Internal P to Grain P Nutrition

The amount of P in grains of low P plants was 2.4-fold lower than in high P plants. This difference was not due to a grain dilution effect since there was no significant difference in grain yield between high and low P plants (**Table 1**). Our previous research with the same cultivar have shown that durum wheat plants can rely on P remobilization to sustain grain growth if they were well supplied with P during vegetative growth (El Mazlouzi et al., 2020). In addition, P can be remobilized from all plant organs including roots, and it can also be stored in both inorganic and organic forms veneklaas (Veneklaas et al., 2012). When the growth requirements are satisfied, plants can store and accumulate further nutrients in their organs for later use (Chapin et al., 1990). Lauer et al. (1989) showed that storage P was remobilized to a greater extent than metabolic P in hydroponically grown soybeans. In other species (e.g., Proteaceae), the remobilization of P can reach 85% of total P in senescent leaves (Veneklaas et al., 2012; Lambers et al., 2015). In the present study, the high P plants had a higher P pool to be remobilized to sustain grain growth and filling whereas low P plants needed to remobilize metabolic P from different plant organs. This may explain the visual accelerated senescence in this treatment (**Figure 2**). The up-regulation of enzymes such as RNases and acid phosphatases that are involved in the breakdown of organic P (e.g., P-esters) has also been shown to increase during leaf senescence in many species (Veneklaas et al., 2012; Jeong et al., 2018). Grains are the last sink accumulating P in wheat. Processes involved in the accumulation of P in grains include P remobilization from senescing organs, source-sink relationship and P transporters (Wang et al., 2016). Rae et al. (2003) reported that the P transporter Pht1.1 was root specific but Pht1.6 was expressed in flag leaves and played a role in P remobilization into grains in barley. Furthermore, the barley *lpa1-1* gene was identified as a member of the sulphate transporter family, that may be a P transporter involved in P allocation to grains (Ye et al., 2011). Targeting an orthologue of this gene was used to engineer low grain P rice (Yamaji et al., 2017). In this recent study, Yamaji et al. (2017) demonstrated that a node specific transporter, sulphate transporter-like phosphorus distribution (SPDT), was implicated in P distribution through nodes in rice. Knocking out this transporter resulted in a 20% reduction of total P in the grain without any decrease in grain yield.

Although this experiment was carried out in hydroponic conditions, the results obtained indicate that the accumulation of P into grains was essentially *via* remobilization and hardly influenced by post-anthesis P uptake. Low P supply altered the partitioning of the new acquired P within the plant. In high P plants, 56% of P taken up during the post-anthesis had been translocated to grains, but 22% remained in tillers, and the remainder was partitioned between roots and leaves. In contrast, low P plants translocated more than 72% of post-anthesis P uptake to grains and only small proportions were found in roots and tillers (**Figure 3**). Nevertheless, some of this difference in redistribution might be due to the excess levels of P in the high P plants. Our results suggest that durum wheat plants with low P nutritional status regulate tightly the allocation of the newly acquired P to grains. Whether the new acquired P arrives directly in wheat grains is not known. The fine characterization of the processes implicated in P loading to grains would require a short-term labelling experiment. For example, using a ^33^P-tracer in a hydroponic study, Julia et al. (2016) reported that P loading into rice grains involves indirect transfer of P originating from P previously acquired and stored in vegetative organs.

### Lowering Grain P: Implications for P Use and Cycling

To fulfil grain P requirement, the depletion of P from spikelets, leaves and stems resulted in higher P remobilization efficiency in low P plants (**Table 2**). In contrast, a large proportion of the P remained in the vegetative tissues of the high P plants (e.g., post-anthesis tillers, roots and leaves). These findings suggest that reducing P remobilization or the allocation of remobilized P to grains, could lower durum wheat grain P concentrations. Retaining more P in the leaves and other tissues means that less P is available to be transferred into grains. It has been also shown that retaining P in leaves maintains higher photosynthetic capacity in rice (Jeong et al., 2017).

In addition, P nutrition does not only affect plant P metabolism, but also could influence the uptake, transport and storage of other nutrients, especially micro-nutrients such as zinc (Zn) and iron (Fe) (Briat et al., 2015). In the particular case of cereals intended for human and feed consumption, lowering P loading into grains is beneficial because the bioavailability of Zn and Fe might also be improved in grains with low P concentrations (Brinch-Pedersen et al., 2002). Phytate, the storage form of P in grains, has a strong capacity in chelating these elements (Raboy, 2009). Low phytate crops are considered a promising strategy to increase grain quality and optimize P balance in farming systems (Raboy, 2009). One successful example of a low phytate crop that reached the field is the barley *low phytic acid 1-1* (Bregitzer and Raboy, 2006). This genotype has a 10 to 15% reduction in grain total P and 50% of phytic acid but no yield reduction as compared with the wild type (Bregitzer and Raboy, 2006; Raboy et al., 2015). A similar result was also obtained for soybean where large reductions in seed phytic acid content had no detectable effect on seed germination and early seedling growth (Raboy et al., 1985). Lott et al. (2000) estimated that the amount of P in phytate is equal to nearly 65% of the global annual P applied as fertilizer. Consequently, grain P has value as a target in P management at both regional and global scales. How theses nutrients are transported to grains and the mechanisms of regulation are not yet fully characterized. One major knowledge gap remains to know whether the P allocated to grains comes from direct fluxes (root to grain *via* xylem transport) or indirect P fluxes (*via* phloem loading). A better understanding of these relationships is important to determine which trait to prioritize in genetic improvement programs, and could help improve the food quality while optimizing crop P uptake and use.

## Conclusion

We investigated the effect of P supply on the partitioning of post-anthesis P uptake and the contribution of internal P sources to grain P nutrition in durum wheat cv. *Sculptur*. Our findings indicate that a large proportion of grain P originates from the remobilization of internal P even at high external P supply during grain filling. The enhanced remobilization of P and the efficient allocation of newly acquired P to grains were crucial to provide the grains with P under low P supply. Nevertheless, the relative increase in the remobilization of pre-anthesis P stores when the plant P status was low, indicates that these fluxes are inter-dependent with the post-anthesis P uptake. For a fine characterization of the processes implicated in P loading to grains, experiments in our laboratory are underway to determine the fate of the post-anthesis P before reaching the grain using a short-term ^32^P tracer approach. Understanding the mechanisms that regulate P loading into grains might help prioritize routes for the improvement of more P-efficient crops.

## Data Availability Statement

All datasets presented in this study are included in the article/**Supplementary Material**.

## Author Contributions

ME and AM conceived and designed the experiments. ME, CC, TR, and AM performed experiments and collected the data. ME, CM, and AM analyzed the data. ME wrote the original manuscript. CM and AM revised the manuscript. All authors contributed to the article and approved the submitted version.

## Funding

This work was supported by funding from the French National Institute for Agriculture, Food and Environment (INRAE), Bordeaux Sciences Agro and the University of Bordeaux.

## Conflict of Interest

The authors declare that the research was conducted in the absence of any commercial or financial relationships that could be construed as a potential conflict of interest.
