# Supplementary material for: Contribution of External and Internal Phosphorus Sources to Grain P Loading in Durum Wheat (Triticum durum L.) Grown Under Contrasting P Levels
Source: Front Plant Sci. 2020 Jun 18;11:870. doi: 10.3389/fpls.2020.00870 (PMC7314961; doi:10.3389/fpls.2020.00870)
Supplement: Supplementary file 1 [file DataSheet_1.pdf]

Supplementary Material

1 SUPPLEMENTARY FIGURES

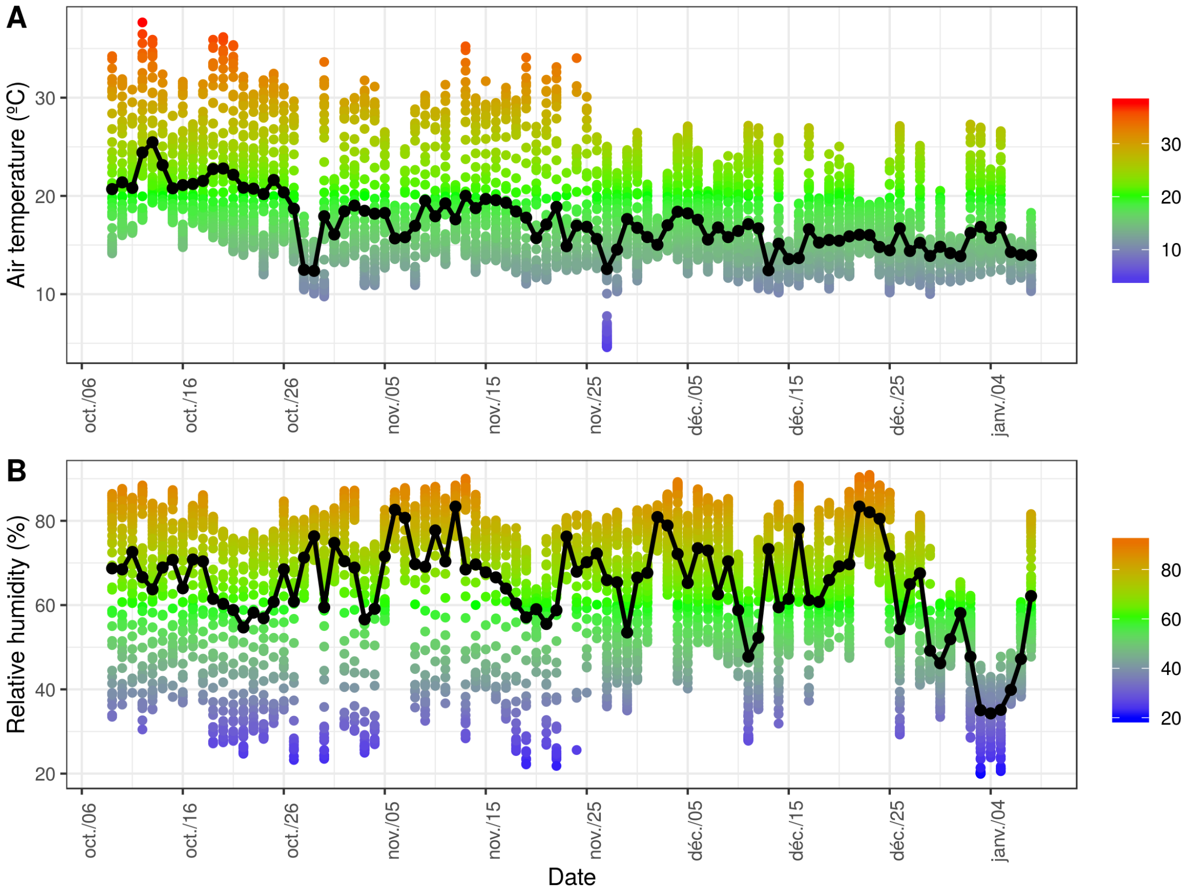

Figure S1. Plant growth conditions in the greenhouse. Air temperature (A) and relative humidity (B) during the experiment. For each measurement mean is shown in black line.
